# Beyond Emotions: Oscillations of the Amygdala and Their Implications for Electrical Neuromodulation

**DOI:** 10.3389/fnins.2019.00366

**Published:** 2019-04-18

**Authors:** Lisa-Maria Schönfeld, Lars Wojtecki

**Affiliations:** ^1^Comparative Psychology, Institute of Experimental Psychology, Heinrich Heine University Düsseldorf, Düsseldorf, Germany; ^2^Institute of Clinical Neuroscience and Medical Psychology, Medical Faculty, Heinrich Heine University Düsseldorf, Düsseldorf, Germany; ^3^Department of Neurology, Center for Movement Disorders and Neuromodulation, Medical Faculty, Heinrich Heine University Düsseldorf, Düsseldorf, Germany; ^4^Department of Neurology and Neurorehabilitation, Hospital zum Heiligen Geist, Kempen, Germany

**Keywords:** amygdala, basal ganglia, neuromodulation, deep brain stimulation, oscillations

## Abstract

The amygdala is a structure involved in emotions, fear, learning and memory and is highly interconnected with other brain regions, for example the motor cortex and the basal ganglia that are often targets of treatments involving electrical stimulation. Deep brain stimulation of the basal ganglia is successfully used to treat movement disorders, but can carry along non-motor side effects. The origin of these non-motor side effects is not fully understood yet, but might be altered oscillatory communication between specific motor areas and the amygdala. Oscillations in various frequency bands have been detected in the amygdala during cognitive and emotional tasks, which can couple with oscillations in cortical regions or the hippocampus. However, data on oscillatory coupling between the amygdala and motor areas are still lacking. This review provides a summary of oscillation frequencies measured in the amygdala and their possible functional relevance in different species, followed by evidence for connectivity between the amygdala and motor areas, such as the basal ganglia and the motor cortex. We hypothesize that the amygdala could communicate with motor areas through coherence of low frequency bands in the theta-alpha range. Furthermore, we discuss a potential role of the amygdala in therapeutic approaches based on electrical stimulation.

## Introduction

The amygdala is one of the core regions associated with emotions and has gained broad interest for its role in emotional conditioning, especially fear conditioning (LeDoux et al., 1990). Besides conditioned fear responses, the amygdala plays an essential role in context-based acquisition of fear responses, PTSD, social anxiety and preparing the organism to react upon a threat (Phillips and LeDoux, 1992; Killgore and Yurgelun-Todd, 2005; Alvarez et al., 2008; Morey et al., 2012; O’Doherty et al., 2017; Engelen et al., 2018). Furthermore, the amygdala is receiving additional attention for its modulating role in social behavior, learning processes, addiction and mood disorders (Langevin, 2012; Bickart et al., 2014; Janak and Tye, 2015). Several human studies also imply an influence of the amygdala on motor and autonomic responses. Bilateral amygdala lesions led to impaired recognition of fearful faces due to an inability of gaze fixation on the eyes (Adolphs et al., 2005; Kennedy and Adolphs, 2010). Also the amygdala is responsible for defensive behaviors in response to acute threats (Klumpers et al., 2017) and increased connectivity between the amygdala and cortical regions is predictive of heart rate variability in patients suffering from generalized anxiety disorder (Makovac et al., 2016). Abnormal functioning of the amygdala is observed in various psychiatric disorders such as generalized anxiety disorder, PTSD, specific phobias, depression, autism spectrum disorder and antisocial personality disorder (Baron-Cohen et al., 2000; Ferri et al., 2017; Fonzo and Etkin, 2017; Garcia, 2017; Kolla et al., 2017; Henigsberg et al., 2019). However, the amygdala might also play a role in psychiatric symptoms of movement disorders, as smaller amygdala volumes are linked to anxiety symptoms in early PD Vriend et al. (2016).

Parkinson’s disease is a movement disorder that can be treated with DBS as stimulation of the STN, the internal GP_i_, or the VIM can reduce core motor symptoms, such as hypokinesia, rigor, tremor, and dyskinesias, in patients that do not benefit from pharmacotherapy alone (Moldovan et al., 2015). In PD beta oscillatory neural activity is observed in the dorsolateral motor part of the STN, which is thought to be a mediator for motor symptoms associated with the disease (Accolla et al., 2016). DBS in PD is assumed to interfere with the pathological oscillations in the basal ganglia by superimposing another oscillatory stimulation pattern with a higher frequency, which paradoxically stops the entrainment of neurons to the pathological frequency and allows them to fire at a more irregular pattern (Wilson, 2014). For PD patients that are not suited to receive DBS, MCS, delivered via flat electrodes positioned epi- or subdurally, might pose an alternative treatment option (De Rose et al., 2012) although its effectiveness in PD is unclear (Tsubokawa et al., 1991; Moro et al., 2011; De Rose et al., 2012).

Besides severe motor disabilities, psychiatric symptoms such as depression, psychosis and anxiety often co-occur and severely impact the quality of life of PD patients (Aarsland et al., 1999). The effect of DBS treatment on non-motor symptoms of PD is not fully explored yet and there are indications that STN-DBS might lead to a significant reduction in anxiety (Witt et al., 2008; Fabbri et al., 2017). However, DBS treatment in itself can cause non-motor side effects, such as transient depressive episodes, pathological crying, laughter or mania (Bejjani et al., 1999; Krack et al., 2001; Herzog et al., 2003; Wojtecki et al., 2007). These side effects are assumed to occur either by stimulating the limbic connections of the STN (Wojtecki et al., 2007) or by current spread from the STN to neighboring areas. One of these areas might be the amygdala; however, current spread to other regions, such as the cingulate cortex might contribute to non-motor side effects as well.

Although there is evidence for structural and functional connections between the amygdala and motor areas, such as the basal ganglia or the motor cortex, premotor cortex and supplementary motor cortex (Peron et al., 2016; Markovic et al., 2017; Loonen and Ivanova, 2018), studies on oscillatory communication between motor areas and the amygdala are lacking. The aim of this review is first, to collect evidence that might hint toward oscillatory coupling of the amygdala with the basal ganglia and the motor cortex. In addition, potential frequency bands are proposed through which communication between regions could occur. Second, it will be discussed whether modulation of amygdala activity has any therapeutic relevance to treat movement disorders. Modulation of the amygdala could most likely occur through indirect stimulation via connected brain regions or even through DBS of the amygdala itself.

## Oscillations in the Amygdala

Neural activity can fluctuate at a periodic interval, which leads to oscillations with a specific frequency and amplitude (Buzsaki, 2006). Oscillations can occur locally within a single brain region, but also synchronized between two or more brain regions. This “coupling” of oscillations is assumed to reflect information processing (Schnitzler and Gross, 2005) and arises through synchronizing oscillations in several ways. Neuronal populations can couple within a distinct frequency by oscillating in-phase, which can be quantified using coherence as a measure. Furthermore, coupling can also be calculated between two different frequencies (cross-frequency coupling). Classically, five categories of oscillation frequencies are defined: delta (0.5–4 Hz), theta (4–8 Hz), alpha (8–12 Hz), beta (12–30 Hz), and gamma (above 30 Hz; Buzsaki, 2006). The frequency specific functions of brain oscillators are complex. As Buzsaki and Draguhn (2004) explain with reference to multiple literature sources: “However, different oscillatory classes might carry different dimensions of brain integration [… ]. Slow rhythms synchronize large spatial domains and can bind together specific assemblies by the appropriate timing of higher frequency localized oscillations [… ].” In other words: “Higher frequency oscillations are confined to a small neuronal space, whereas very large networks are recruited during slow oscillations [… ]. This relationship between anatomical architecture and oscillatory pattern allows brain operations to be carried out simultaneously at multiple temporal and spatial scales [… ].” Besides that, a region specific or function specific interpretation of frequency bands is empirically described in various publications. In this review we list some of these findings of oscillations related to the amygdala.

A small number of human studies have been conducted where oscillations in the amygdala were measured. PTSD patients received fMRI-based neurofeedback and a successful lowering of alpha frequencies co-occurred with a shift in connectivity of the BLA from fear and memory-related structures, such as the hippocampus and the periaqueductal gray, toward prefrontal areas involved in emotion regulation (Nicholson et al., 2016). Oscillations in the alpha range have been associated with selective attention and information processing (for review see Klimesch, 2012). Furthermore, alpha desynchronization during a therapeutic session enabled connectivity changes between brain regions (Ros et al., 2013), which might lead to decreases in oscillatory power in specific regions such as the amygdala. Epilepsy patients with electrodes implanted in the BLA and the hippocampus exhibited high gamma oscillations (70–180 Hz) in both regions upon viewing fearful faces. Moreover, gamma oscillations in the BLA preceded those in the hippocampus (Zheng et al., 2017). An entrainment of hippocampal gamma oscillations by the BLA might suggest a modulatory role of the BLA in the processing of fearful stimuli and retrieval of fearful memories.

In contrast to the sparse number of human studies, several animal experiments have been performed that led to mechanistic insights about oscillations in the amygdala. Consolidation and retrieval of fearful memories in mice led to theta oscillatory coherence between the BLA and the hippocampus (Seidenbecher et al., 2003; Narayanan et al., 2007). In the hippocampus theta oscillations are associated with memory consolidation and retrieval (Hasselmo, 2005) and again, its increased communication with the BLA might point at enhanced processing of emotional memories as opposed to more neutral ones. In the case of fear extinction learning in mice, interneurons in the BLA play a pivotal role, since they enable oscillations in the alpha range (here 6–12 Hz), which interfere with fear-associated oscillations in the low theta range (here 3–6 Hz; Davis et al., 2017). Increases in theta power in the BLA have also been measured while mice entered a non-social compartment instead of a social compartment in a spatial decision task. Interestingly, injections of the NMDA receptor antagonist Ketamine abolished differences in theta power when entering the compartments (Mihara et al., 2017). In another experiment, DBS in the infralimbic cortex in anesthetized rats caused increases in slow wave (<1.5 Hz), theta and fast gamma power in the BLA, coupling between slow waves with faster theta and beta frequencies within the BLA and coherence of slow waves and theta frequencies between the BLA and the hippocampus (Cervera-Ferri et al., 2016). The increase in oscillatory activity in the BLA and the enhanced coherence between the BLA and the hippocampus upon infralimbic cortex DBS might be relevant for the therapeutic effect of cingulate gyrus DBS. The cingulate gyrus in humans corresponds to the infralimbic cortex in rodents and DBS of the cingulate gyrus has been shown to reduce depressive symptoms (Uylings et al., 2003; Mayberg et al., 2005). During reward learning in cats, increased coherence of gamma oscillations between the BLA and rhinal cortices was observed, which was initiated by enhanced synchrony of BLA neurons (Bauer et al., 2007). In the mouse BLA, fast gamma oscillations were coupled to theta waves during states of fear, whereas coupling decreased during states of safety (Stujenske et al., 2014). Simultaneously to this decrease in local theta-gamma coupling during safety, gamma power increased in the BLA and a stronger coherence between the BLA and the mPFC was detected, which might reflect a suppression of learned feelings of fear (Stujenske et al., 2014). Avoidance of shock delivery to a fellow rat was linked to low gamma coherence between the insula, OFC and BLA, whereas choices, which resulted in another rat receiving a shock, were linked to increased gamma coherence between the same regions. Interestingly, high gamma oscillations in the BLA preceded those in the insula (Schaich Borg et al., 2017). In addition, stronger alpha power was observed in several brain regions, including the amygdala, and correlated positively with avoidance of shock delivery. Within the network of interest, the amygdala seemed to be the source of alpha oscillations as its activity preceded alpha oscillations measured in other brain areas (Schaich Borg et al., 2017). Theta oscillations that have been observed in rats in a social fearful context (Tendler and Wagner, 2015) were measured in the BLA and correlated negatively with the shock avoidance behavior described above (Schaich Borg et al., 2017).

## Connections of the Amygdala With Motor Areas and Implications for Electrical Neuromodulation

Several studies using tracing or imaging techniques have discovered structural connections between the amygdala and different motor areas in humans and animals. In this section “motor areas” encompass the STN, GP and the motor cortex, as these are the main clinically relevant targets to treat movement disorders such as PD or Huntington’s disease; however, the amygdala also projects to other motor areas that are beyond the scope of this review.

In humans, diffusion tensor imaging provided evidence for structural connectivity between the dorsal part of the amygdala and the motor cortex through the external capsule, which grants the amygdala a significant influence on motor behavior (Grezes et al., 2014). More recently, the existence of a functional circuit between the amygdala and the sensorimotor cortex at rest was demonstrated in an fMRI study using a large number of participants, further supporting the assumption that the amygdala is a critical modulator of motor behavior (Toschi et al., 2017). Other imaging studies revealed functional connectivity between the STN and the amygdala (Peron et al., 2016) and the GP and the amygdala (Yang et al., 2017).

Tracing studies in animals have provided more detailed insights about anatomical links between the amygdala and motor regions by revealing monosynaptic connections between both. Primate and rodent studies have detected structural connections between the amygdala and the motor cortex (Kita and Kitai, 1990; McDonald, 1998). Anterograde tracing in rhesus monkeys showed projections from the BLA onto the motor cortex, the majority of which terminated in the face and arm representation (Morecraft et al., 2007). The latter finding is interesting, considering the role of the amygdala in processing and expressing emotions. Recently, projections from neurons positive for corticotropin-releasing factor in the central amygdala to the GP_e_ were discovered, indicating a novel circuit for stress-relevant information (Hunt et al., 2018). In rats, high frequency stimulation of the STN caused an increase in neural activity in the BLA (Hachem-Delaunay et al., 2015); however, proof of direct structural connections between the amygdala and the STN remains absent.

The connections between the basal ganglia and the motor cortex including their alterations in a typical movement disorder such as Huntington’s disease have been described in detail (Wojtecki et al., 2016). Upon receiving cortical input, the striatum projects either directly to the GP_i_ and the SNr (*direct pathway*) or indirectly via the GP_e_ and the STN (*indirect pathway*). The GP_i_ and SNr in turn send inhibitory projections to the thalamus that provides excitatory input to the motor cortex (Calabresi et al., 2014). Moreover, projections from cortical layer V are assumed to target the STN via the *hyperdirect pathway*. These projections might originate in the primary motor cortex or neighboring cortical regions like the supplementary motor area and the dorsal and ventral divisions of the premotor cortex (Nambu et al., 2002). Interestingly, stimulation of cortical layer V neurons reduced Parkinsonian symptoms in mice (Gradinaru et al., 2009), indicating that the motor cortex might play a causal role in the pathogenesis of PD and that DBS of the STN might be effective through antidromic activation of the motor cortex via the hyperdirect pathway (Arbuthnott and Garcia-Munoz, 2017). The amygdala has the potential to influence all three pathways within the basal ganglia due to its connections to the STN, the GP and the motor cortex. In fact, it was recently shown that PD patients who suffer from freezing of gait have higher resting state connectivity between the amygdala and the striatum compared to PD patients that do not suffer from freezing of gait (Gilat et al., 2018), indicating that abnormal amygdala activity worsen the clinical picture of movement disorders.

### Potential Oscillatory Interactions Between the Amygdala and Motor Regions

Abnormal amygdala activity has been associated with anxiety disorders for decades and recently became acknowledged for its role in pathological motor symptoms (Gilat et al., 2018). Due to connections with motor areas that are frequent targets of DBS, modulation of the amygdala might occur indirectly as a result of network changes induced by the stimulation and could account for emotional side effects that arise from DBS. Side effects linked to DBS of the STN include worsening of verbal fluency, cognitive deterioration, hypomania and impairment in affect regulation (Krack et al., 2003; Wojtecki et al., 2007; Witt et al., 2008; Kim et al., 2012). The STN and presumably also the GP_e_ have direct functional connections to the amygdala and a therapeutic alteration of oscillations within these nuclei could change the input to the amygdala, inducing potentially unwanted effects on learning, memory and emotions. Pathological beta oscillations in the STN are a major hallmark of PD and can be suppressed by DBS (Quinn et al., 2015). A decrease in these beta oscillations and an increase in theta-alpha power in the STN are associated with superior motor performance of PD patients (Anzak et al., 2012; Tan et al., 2013). Also an increase in theta-alpha frequencies in the STN during a verbal generation task was found with increased coherence between the STN and frontal cortical association areas (Wojtecki et al., 2017). However, theta oscillations in the amygdala have frequently been reported to co-occur with states of fear and anxiety (Narayanan et al., 2007; Davis et al., 2017). If theta oscillations by the STN would induce theta oscillations in the amygdala following DBS, improved motor performance might come with the cost of increased anxiety.

In the basal ganglia of PD patients, alpha frequencies are observed and while some researchers report an influence of dopaminergic medication (Gironell et al., 1997), others did not show any medication-induced change in alpha frequencies (Brittain and Brown, 2014). Furthermore decreases in alpha frequencies have been linked to PD-associated dementia (Cozac et al., 2016). In a rat study, the BLA has been the source of alpha oscillations during social decisions, entraining connected regions (Schaich Borg et al., 2017). Alpha frequency entrainment of motor regions by the amygdala would be an interesting mechanism to improve cognitive functions in PD patients suffering from dementia. However, inductions of oscillations at a specific frequency in the amygdala might require direct electrical stimulation, which has many drawbacks as discussed in the following section.

Beta frequencies represent the majority of oscillations in the motor cortex and were coupled to delta frequencies in a motor task that specifically requires attention and planning (Saleh et al., 2010). As the amygdala provides direct input to the motor cortex, it is also closely involved in the generation of motor behavior. Thereby, amygdala modulation might cause disturbances in psychomotor functioning, which have been observed in the form of pathological crying that occurred in the absence of adequate emotions (Wojtecki et al., 2007). Still, the exact role of the amygdala in the occurrence of non-motor side effects during DBS needs further investigation.

**FIGURE 1:**
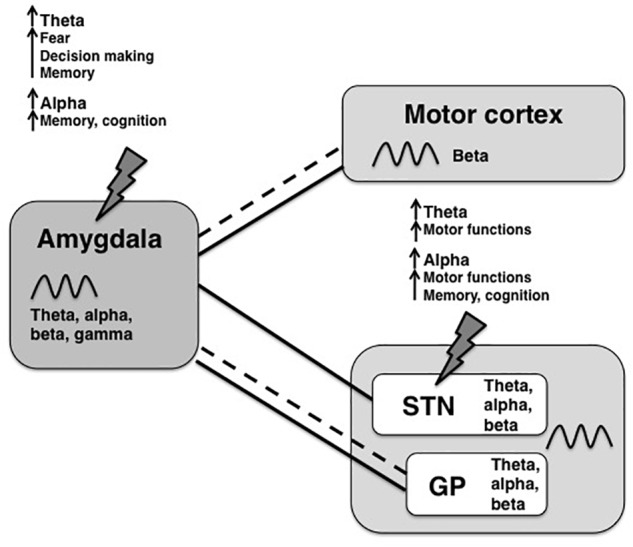
Overview of oscillatory frequencies and connections between amygdala and motor areas. The amygdala is structurally (dashed lines) and functionally (solid lines) connected to motor areas, such as the motor cortex, the limbic part of the subthalamic nucleus (STN) and the globus pallidus (GP). For all shown regions, a broad spectrum of oscillation frequencies has been previously described, but the frequency bands that are used for information transfer between the amygdala and the basal ganglia are not known yet. In this review, we propose coherence of theta-alpha oscillations as a possible way of inter-regional communication with potential therapeutic value. Upon stimulation of the amygdala-motor circuit, for example by STN-DBS or amygdala-DBS (indicated by lightning bolts), theta and alpha frequencies might be increased within the circuit, where each frequency has different functional properties depending on the source region.

To reduce non-motor side effects of DBS, theta and alpha frequencies would be interesting candidate frequencies to modulate within an amygdala–basal ganglia network. Theta and alpha frequencies in the STN have been associated with improved motor functions in PD patients (Anzak et al., 2012; Tan et al., 2013) and have been observed during focused attention, potentially having a gating function in decision-making (Cavanagh et al., 2011; Wojtecki et al., 2017). Although theta oscillations in the BLA have been associated with fear responses (Davis et al., 2017), increases in theta power were measured also during social decision tasks, specifically when animals chose against the social option (Mihara et al., 2017; Schaich Borg et al., 2017). The latter might reflect a rather general, emotionless role of theta oscillations in choice behavior since increases in theta power have been linked to novel stimuli (Tendler and Wagner, 2015). Alpha oscillations are found in both the basal ganglia and the amygdala and are thought to act as an inhibitory filter in attention processes (Klimesch, 2012) and might have the potential to improve cognition. Lastly, slow theta-alpha frequencies might reflect information transfer over long distances across the brain (von Stein and Sarnthein, 2000), which makes them suitable of facilitating communication between the amygdala with the basal ganglia and the cortex. A simplified summary of connections between the amygdala and motor areas, the most common oscillatory frequencies found in each region and hypothetical shared oscillations are provided in Figure 1. In that scheme slower rhythms such as theta or alpha might synchronize the activity of distal neuronal networks (e.g., between the amygdala and the basal ganglia) and couple to local higher frequencies (for ex., gamma within the amygdala).

### Amygdala DBS: Worth the Risk?

Electrical stimulation of the amygdala possesses several potential obstacles, which make it a risky, if not unsuitable target for DBS in humans. First, segregating the specific nuclei within the amygdala is not possible with standard MRI-based localization approaches and amygdala anatomy varies substantially across subjects (Oya et al., 2009). Second, the amygdala is a known kindling site in animal studies, where electrical stimulation can lead to recurring after-discharges and seizures (Cleeren et al., 2016; Li et al., 2016; Wicker and Forcelli, 2016). In addition, structural changes occur in patients suffering from temporal lobe epilepsy (Reyes et al., 2017), which further complicates target localization at least in these patients.

Due to the associated risks, DBS of the amygdala has only been performed in a few cases. Seizure frequency could be reduced by amygdala DBS in patients suffering from refractory temporal lobe epilepsy, without causing behavioral alterations or stimulation-induced seizures (Vonck et al., 2002; Boon et al., 2007). Furthermore, brief electrical stimulation during a cognitive task improved declarative memory, potentially by increasing oscillations in the theta and gamma band between the amygdala, the hippocampus and the perirhinal cortex (Inman et al., 2018). DBS of the BLA in a child suffering from autism caused a decrease in self-directed aggression and improvements in cognitive, social and emotional functioning (Sturm et al., 2012). The BLA might be seen as a communication hub between different centers, for example the frontal cortex, sensory cortices and the basal ganglia, that are all involved in the generation of autistic symptoms and the improvement in symptoms could have been achieved by a reset of oscillatory activity in the BLA (Sinha et al., 2015). In a PTSD patient intraoperative microelectrode recordings showed an increased firing of the right BLA in the beta range, whereas predominant frequencies in the left BLA were in the theta-alpha range. DBS of the BLA led to a reduction of anxiety and improvements in sleeping pattern (Langevin et al., 2016). In PTSD patients, the amygdala is overactive in response to trauma-related stimuli, but also during unrelated emotional tasks and at rest (Reznikov and Hamani, 2017) and DBS might normalize its activity. Given the potential of amygdala DBS to treat PTSD, a larger clinical trial has been proposed, but results are not published yet (Koek et al., 2014).

A few studies have been conducted to specifically assess potential emotional alterations following amygdala stimulation, with a diverse outcome. Unilateral amygdala stimulation caused an increase in feelings of fear, regardless which hemisphere was stimulated (Meletti et al., 2006). Conversely, stimulation of the right BLA in a single case created a positive affective bias during the evaluation of emotional stimuli (Bijanki et al., 2014). In another study, stimulation of the right amygdala was associated with increased feelings of fear and sadness, whereas stimulation of the left amygdala could evoke both pleasant (happiness) and unpleasant (fear, sadness, and anxiety) states (Lanteaume et al., 2007).

Taken together, the body of evidence for DBS to treat psychiatric symptoms is growing, but given the risk associated with amygdala DBS and its potential to evoke a variety of negative emotions, this target needs further thorough investigation.

## Discussion

The amygdala is connected to brain regions such as the hippocampus, the ventral striatum and the cortex; therefore, it is embedded in a network involved in generating emotions and learning (Langevin, 2012; Gibson et al., 2017). Structural connections between the amygdala and motor areas, such as the STN, GP and motor cortex have been discovered in different animal species (Morecraft et al., 2007) and resting state connectivity between the amygdala and the basal ganglia has been shown recently in a subpopulation of PD patients (Gilat et al., 2018). In addition, stimulation of different subregions belonging to the cingulate cortex, which are connected to the amygdala, induced emotional motor responses (Caruana et al., 2015, 2018). In a recent study it was demonstrated that emotional stimuli induced an effect on motor behavior, potentially mediated by connections from the amygdala to motor areas. Interestingly, that behavioral effect was only evident when the emotional content of the stimuli was task-relevant (Mirabella, 2018).

Up to date, no simultaneous electrophysiological measurements have been performed in the amygdala and motor areas. Based on structural connections between the amygdala and motor areas, we raised the question whether amygdala modulation could potentially alleviate psychiatric symptoms of PD or conversely could induce non-motor side effects that occur during DBS of the basal ganglia. Modulation of the amygdala might cause or exacerbate some non-motor side effects that occur during DBS of the basal ganglia, for example by alterations in theta and alpha frequencies. On the contrary, DBS of the BLA has been shown to decrease anxiety (Langevin et al., 2016), potentially by decreases in theta power, therefore direct or indirect modulation of the amygdala could support the management of anxiety associated with PD. As there is scarce feasibility of amygdala DBS without negative side effects it is worthwhile to consider non-invasive stimulation techniques, such as tDCS or TMS for indirect neuromodulation of the amygdala through a cortical network entry point. These techniques can induce short term or even chronic alterations in neuronal activity without the risk associated with implanted stimulation electrodes. However, these techniques only allow precise stimulation of superficial cortical regions. To gain insight into possible mechanisms of such an indirect amygdala modulation it is relevant to study amygdala oscillatory coupling with motor cortical regions.

Taken together, there is evidence for structural connections between the amygdala and motor areas, such as the basal ganglia and the motor cortex, that are functionally altered in movement disorders. We hypothesized that information transfer between the amygdala and motor areas occurs through oscillations in the alpha and theta frequencies, potentially having either beneficial or adverse effects. In the future, simultaneous electrophysiological measurements in the amygdala and motor regions are needed to specify oscillatory connections during cognitive and motor tasks. To obtain reliable behavioral effects, task-relevance of stimuli seems to be important, which should be investigated in relation to the amygdala and motor areas. Based on these findings, oscillation frequencies that show coherence between the amygdala and motor areas could be specified to prevent psychiatric side effects of DBS or even use secondary amygdala activation as an add-on for current DBS or MCS therapy.

## Author Contributions

L-MS wrote the first draft, which was revised by LW. Both authors conceptualized the topic and content of the review, and approved the manuscript.

## Conflict of Interest Statement

The authors declare that the research was conducted in the absence of any commercial or financial relationships that could be construed as a potential conflict of interest.

## Abbreviations

BLA: basolateral amygdala
DBS: deep brain stimulation
BLA: basolateral amygdala
GP: globus pallidus
GP_e_: external globus pallidus
GP_i_: internal globus pallidus
MCS: motor cortex stimulation
mPFC: medial prefrontal cortex
PD: Parkinson’s disease
PTSD: posttraumatic stress disorder
SNr: substantia nigra pars reticulata
STN: subthalamic nucleus
tDCS: transcranial direct current stimulation
TMS: transcranial magnetic stimulation
VIM: ventral intermediate nucleus of the thalamus
